# 3D FEA of cemented glass fiber and cast posts with various dental cements in a maxillary central incisor

**DOI:** 10.1186/s40064-015-1345-4

**Published:** 2015-10-13

**Authors:** Ahmed A. Madfa, Mohsen A. Al-Hamzi, Fadhel A. Al-Sanabani, Nasr H. Al-Qudaimi, Xiao-Guang Yue

**Affiliations:** Department of Conservative Dentistry, Faculty of Dentistry, University of Thamar, Dhamar, Yemen; Department of Pediatric Dentistry, Preventive Dentistry and Orthodontics, Faculty of Dentistry, University of Thamar, Dhamar, Yemen; Department of Safety Science and Engineering, Wuhan University of Technology, Wuhan, China

**Keywords:** Finite element method, Biomechanics, Dental post, Dental cement, Shear stress

## Abstract

This study aimed to analyse and compare the stability of two dental posts cemented with four different luting agents by examining their shear stress transfer through the FEM. Eight three-dimensional finite element models of a maxillary central incisor restored with glass fiber and Ni–Cr alloy cast dental posts. Each dental post was luted with zinc phosphate, Panavia resin, super bond C&B resin and glass ionomer materials. Finite element models were constructed and oblique loading of 100 N was applied. The distribution of shear stress was investigated at posts and cement/dentine interfaces using ABAQUS/CAE software. The peak shear stress for glass fiber post models minimized approximately three to four times of those for Ni–Cr alloy cast post models. There was negligible difference in peak of shear stress when various cements were compared, irrespective of post materials. The shear stress had same trend for all cement materials. This study found that the glass fiber dental post reduced the shear stress concentration at interfacial of post and cement/dentine compared to Ni–Cr alloy cast dental post.

## Background

Restoration of endodontically treated tooth has been greatly enhanced by many developments in materials and techniques. In many cases, the dental post is often used after root canal treatment to restore a damaged tooth with extensive loss of coronal tooth structure specifically when 50 % or massive of coronal structure have been lost (Kimmel 2000; Ferrari et al. 2001). However, the restoration of endodontically treated tooth, that including dental post, is affected by a higher risk of biomechanical failure than vital tooth (Hatzikyriakos et al. 1992). This is attributable to the relatively high fracture incidence in pulpless tooth (Salis et al. 1987) and the dental post may be displaced during function (Sorensen and Martinoff 1984). Therefore, the dental post should have enough strength to avoid fracture incidence in the remaining tooth structure and it should be cemented with sufficient bonding strength agent to prevent the dislodgment of the dental post during function (Bouillaguet et al. 2003).

Over the past years, post materials and designs have been expanded considerably to rehabilitate the endodontically treated teeth (Ferrari et al. 2000; Brown and Hicks 2002; Schwartz and Robbins 2004). In endodontics, two types of post systems, cast metal dental and prefabricated dental posts, are usually adopted to restore a root canal and provide retention for crown. Although traditional metallic cast post can be performed treatment in one dental visit, they are gradually being replaced by prefabricated posts, particularly those made with glass fiber materials (Bitter and Kielbassa 2007). The benefits for using the glass fiber dental post are aesthetics, better biocompatibility and corrosion resistance (Boschian Pest et al. 2002; Maccari et al. 2003).

Cementation of the dental post into the root canal is critical, because the process should accomplish a seal along the root canal. An ideal luting agent must meet the basic mechanical, biological and handling requisites, such as compatibility to the tooth and tissue, non-toxic, adequate working time, sufficient bonding strength, enough compressive strength, flowability, minimal microleakage, adhesiveness, ease of excess removal, low solubility in oral fluids, esthetics, and low cost (Hill 2007). However, no luting agents have all ideal properties (Creugers et al. 1994). Additionally, many studies showed conflicting in the outcomes of tensile bond strength (Creugers et al. 1994; Mendoza and Eakle 1994; Mitchell 2000). For that reason, there is no consensus in the literature as to the superiority of any cement compared to others.

Zinc phosphate cement is still the luting agent of choice for most conventional dental post restoration, because of its easy handling characteristics and long-term clinical documentation. Recently, resin-based cements were introduced and they showed good adherence to tooth structure. Many studies reported that the resin-based cements showed significantly higher retention and resistance to fatigue compared to zinc phosphate and resin-modified glass ionomer cements (Creugers et al. 1994; Mendoza and Eakle 1994; Mitchell 2000).

As the mechanical properties of the natural teeth differ from materials restoration, the interface between the components represents the critical area for stress concentration. Determination of the stress distributions in endodontically treated tooth restored with dental post is challenged owing to small dimension and complex structure of the post. However, the investigation the stress distribution in the restored endodontically treated tooth with dental post has remained controversial issue (Asmussen et al. 2005; Santos et al. 2009).

**Fig. 1:**
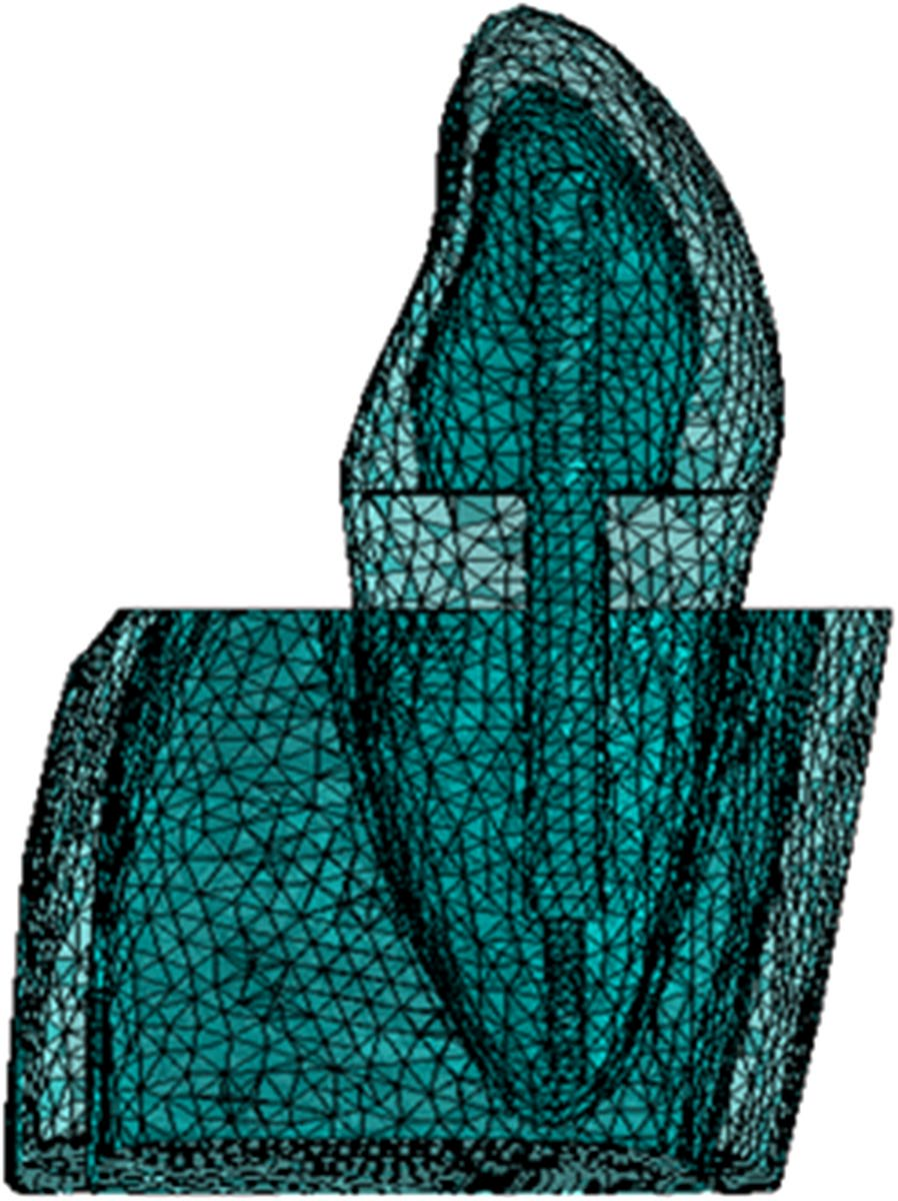
Schematic illustration of the three dimensional geometric model

Finite element method (FEM) has been used by several studies for attempting to know the process of stress dissipation in teeth restored by various restorations (Pegoretti et al. 2002; Boschian Pest et al. 2006; Abu Kasim et al. 2011; Madfa et al. 2014). Therefore, this study aimed to analyze and compare the stability of two dental post materials (Ni–Cr alloy cast and glass fiber) cemented with four different luting materials (zinc phosphate, Panavia™ resin, superbond, C&B resin and glass ionomer) by investigating their shear stress transfer through the FEM (Fig. 1).

## Results

**Fig. 2:**
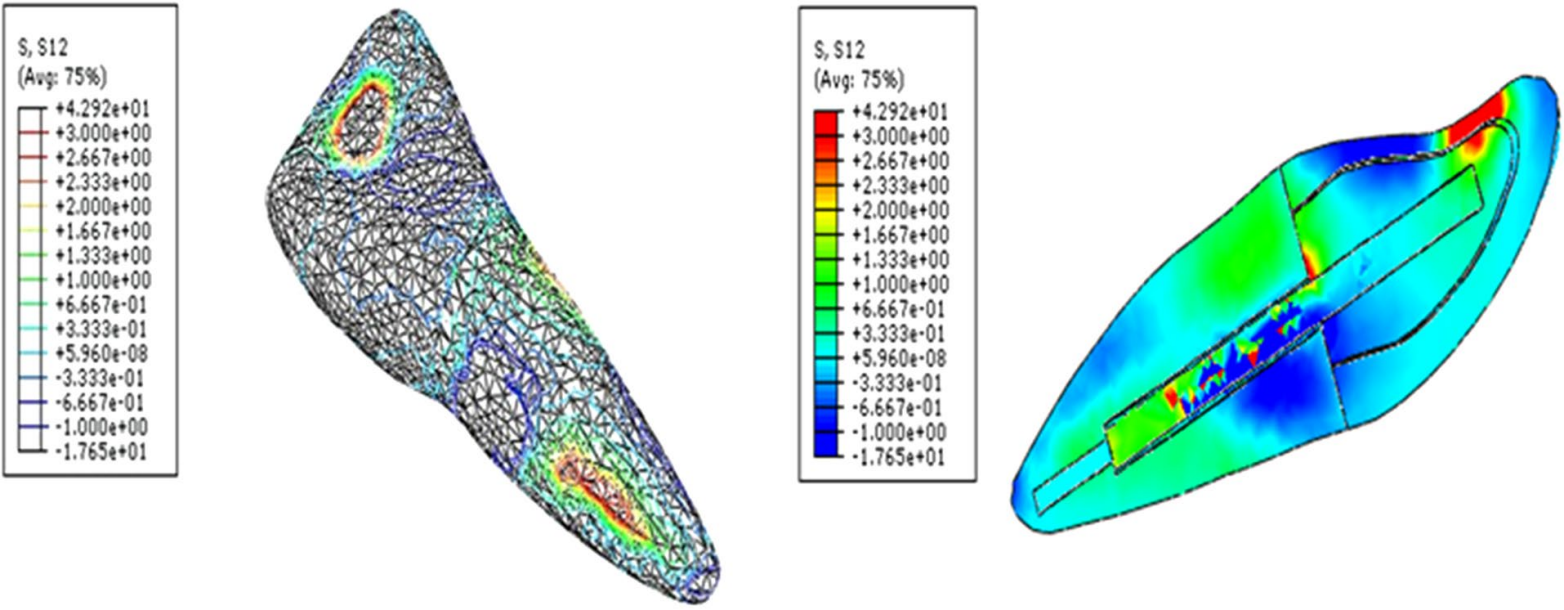
Contour plots of the shear stress distribution in XY direction within the tooth and the posts when loaded

**Fig. 3:**
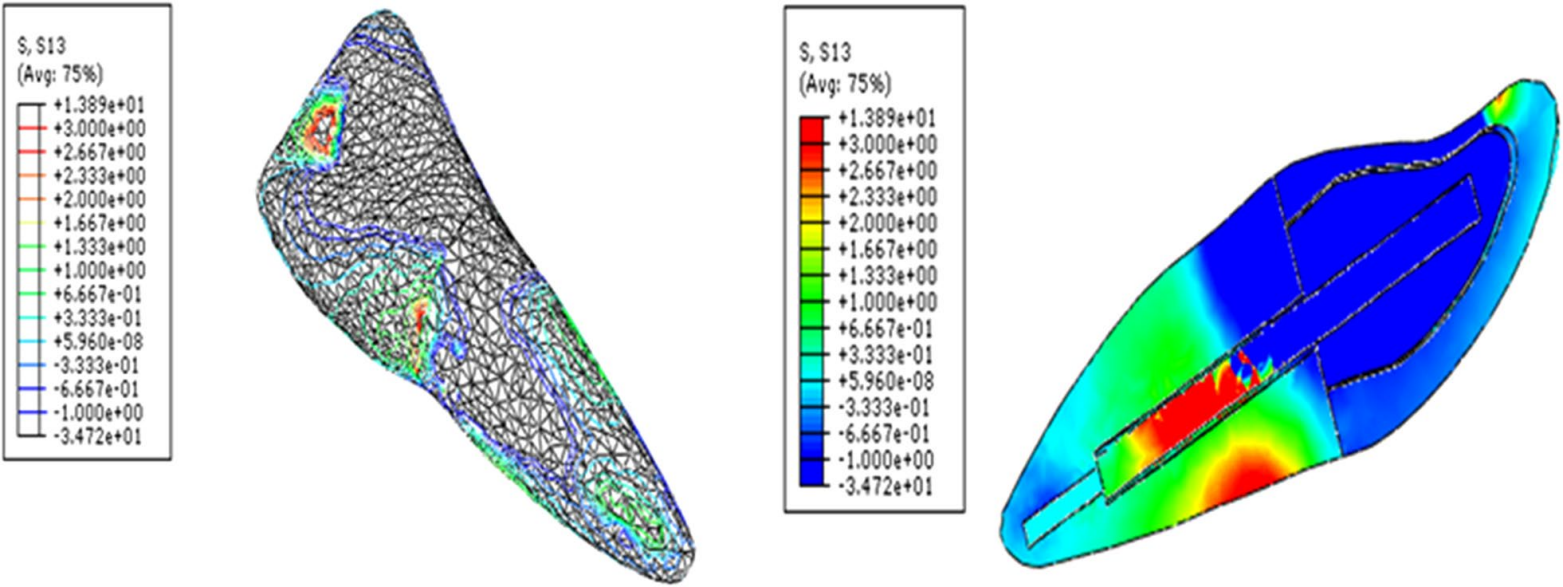
Contour plots of the shear stress distribution in XZ direction within the tooth and the post when loaded obliquely

**Fig. 4:**
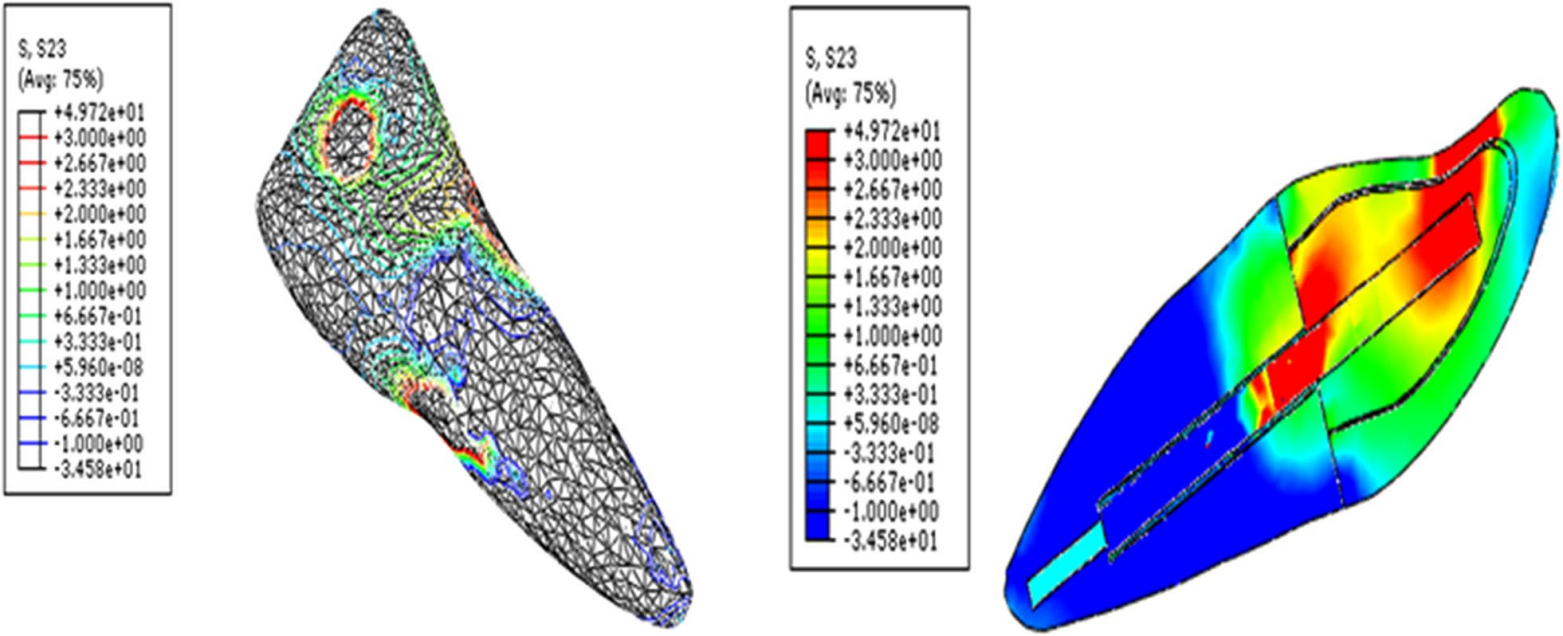
Contour plots of the shear stress distribution in ZY direction within the tooth and the post when loaded obliquely

Shear stress for different post and cement materials following oblique loading are presented in Figs. 2, 3 and 4. Generally, maximum shear stress concentrated at the coronal third of the root (dentine) both on the labial and palatal aspect of the root and also concentrated at apical third of post and cement/dentine interfaces, regardless of post and cement materials.

It was observed that glass fiber dental post introduced lower shear stress compared to Ni–Cr alloy cast post as shown in Table 1. Glass fiber post dissipated interface stress excellently from the coronal to the apical parts of the post and dentine interface. In glass fiber models, the shear stress was considerably diminished in all regions of the interface compared to Ni–Cr alloy cast models.

**Table 1:** The maximum shear stress concentration at the dental post and cement/dentine interface

Post Materials	Ni-Cr custom-made dental post	Glass fiber dental post
XY direction	4.5	0.98
XZ direction	6	2
ZY direction	12	3

The shear stress generated in endodontically restored tooth with glass fiber post was reduced approximately three to four times of those for Ni–Cr alloy cast post, when cement materials was disregarded. The maximum shear stress introduced by glass fiber post was 0.98 MPa (XY), 2 MPa (XZ) and 3 MPa (ZY). However, the maximum shear stress for Ni–Cr alloy cast post were 4.5 MPa (XY), 6 MPa (XZ) and 12 MPa (ZY). There was negligible difference in the peak of shear stresses when various cements were compared, irrespective of post materials. When post materials were disregarded, the shear stress had same trend for all cement materials.

**Fig. 5:**
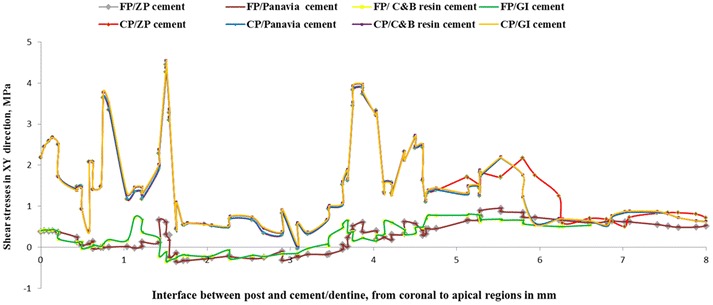
Shear stress distributions in XY direction at the posts and cement/dentine interface

**Fig. 6:**
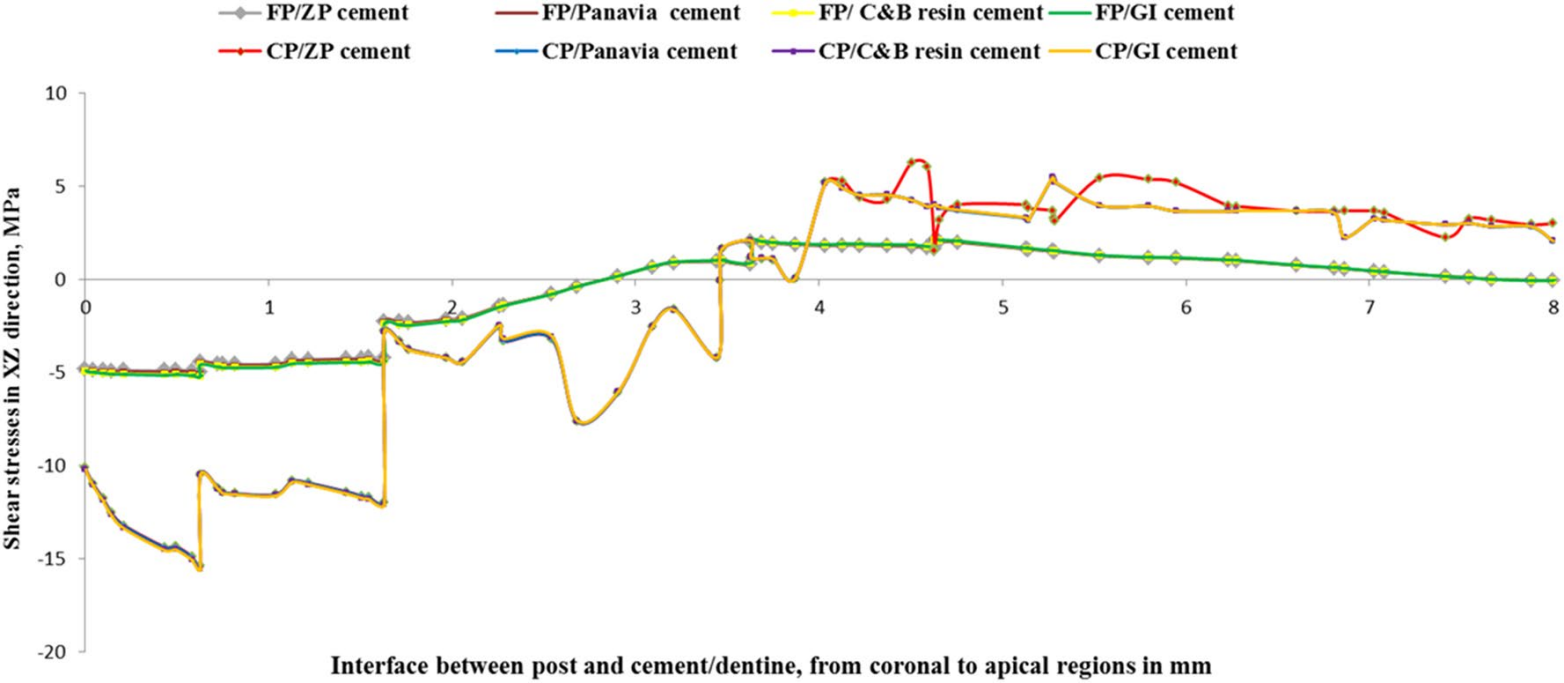
Shear stress distributions in XZ direction at the posts and cement/dentine interface

**Fig. 7:**
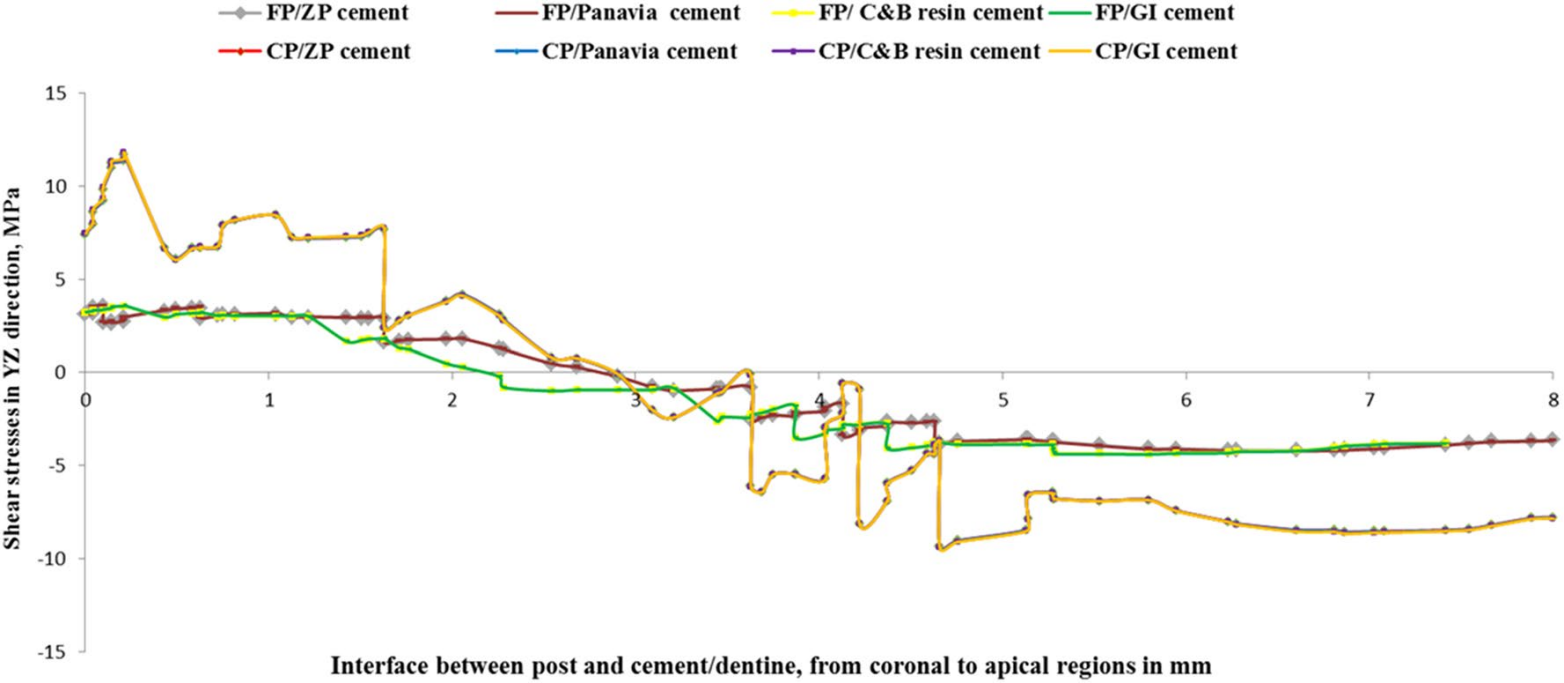
Shear stress distributions in ZY direction at the posts and cement/dentine interface

Two sites of severe interfacial shear stress concentrations were identified. The shear stress was concentrated at the interface of coronal region of root (dentine) and dental post and at the interface of apical region of root (dentine) and post (Figs. 5, 6, 7). This indicates that the interfacial failure initiation should be expected to start from the coronal and the apical regions of the dental post. The transition between positive and negative interfacial stress occurred at middle region of the root where the value of interfacial stress reached zero. Similar trends were obtained among different dental cements as exhibited in Figs. 5, 6 and 7.

## Discussion

The loss of retention for dental posts or root fracture is considered the main reason for biomechanical failure of the restoration (Bergman et al. 1989; Hatzikyriakos et al. 1992; Torbjorner et al. 1995). This study investigated the shear stress transfer at post and dentine/cement interface through the FEM. Calculated shear stresses may be compared to values of adherence obtained with resin based materials (Asmussen and Peutzfeldt 2001) or zinc phosphate cement to assess the risk of loss of retention of the dental post (Drummond et al. 1999). The higher interfacial stress concentration indicates interfacial failure of the restoration (Beer and Johnston 1993). Thus, in the present study, the shear stress was analyzed at three different plans to determine any sign, any pattern, and any location for the maximum values. The stresses analyzed under XY and XZ directions indicated that possibility of displacement of the dental post under the torsion and compression. Therefore, the use of adhesive cementation can contribute to minimize the risk of such displacement. However, stress analysis under ZY direction result in rupture of adhesive cementation at the tooth–post interface and it can therefore lead to loss of retention of the dental post. Thus, in order to increase the clinical lifespan of the restoration inside oral cavity, it is important to reduce stresses in the tooth and the post interface.

Holderegger et al. (2008) and Van Meerbeek et al. (2010) determined the bond strength of dental cements and frequently used to predict clinical behaviour of dental restorations. They reported that most annual failure rates of restorations clinically were highly correlated with the amount of shear bond strength. However, the reported bond strength values showed highly different due to the using various testing methodologies (Van Noort et al. 1989; Placido et al. 2007; Scherer et al. 2010). Additionally, the difficulty in preparing the specimens which, in turn, lead to generate non-uniform stresses within the specimens and then effect significantly on the mode of failure (Scherer et al. 2010).

To overcome these problems, FEM was used to study the shear stress distribution at the dental post and cement/dentine interface. However, accuracy and validity remain substantial challenges. In this study, accuracy was confirmed by a convergence test in which subsequent refinements of the mesh were made (Abu Kasim et al. 2011; Madfa et al. 2014). The second problem, validity, is dependent upon the extent to which the tooth model reflected the real conditions. Therefore, the models used in this study were created from a computed tomography (CT)-scan image of an adult maxillary central incisor and its surrounding structures. This provided a realistic rendition of clinical conditions. The oblique force of 100 N was obtained from the literature evaluating the mechanical behaviour of posts and cores through the FEM (Joshi et al. 2001; Asmussen et al. 2005; Al-Omiri et al. 2011; Madfa et al. 2014).

The primary role of teeth in the oral cavity is to serve as a mechanical device for the mastication of food. The stress distribution pattern in intact natural tooth is different from tooth restored with dental post system. Intact natural tooth can flex or bend during functional load while the tooth restored with dental post system showed occurrence of regions of shear stress concentration at the post and cement/dentine interfaces. This is attributable to that the post system bends or flexes as a single unit during mastication, differences of biomechanical behaviour between remaining reduced tooth structure and dental post system (Eliasson et al. 1995). Additionally, the defective in post and cement/dentine interface could cause shear stress concentration (Pegoretti et al. 2002). The results of this study were in agreement with previous studies that reported that the stress distribution were no uniform and concentrated at post and cement/dentine interfaces (Holmes et al. 1996; Alessandro et al. 2005). They reported that a very stiff post works against the natural function of the tooth creating zones of tension and shear both in the dentine and at the interfaces of the luting cement and the post.

Asmussen and Peutzfeldt (2001) reported that bonding strength of dentine bonding systems was in the range of 15–30 MPa, and posts luted with zinc phosphate cement can be displaced from its place during mastication at a stress level of approximately 5–25 MPa. In this study, the maximum shear stresses were found to be primarily located at the post and surrounding structures interface. The maximum shear stress for the fiber post models was 0.98 MPa (XY), 2 MPa (XZ) and 3 MPa (ZY). However, the maximum shear stresses for Ni–Cr alloy cast post models were 4.5 MPa (XY), 6 MPa (XZ) and 12 MPa (ZY). There was negligible difference in the peak of shear stresses when various cements were compared, irrespective of post materials. This is attributable to that the stiffness of the dental cements used in this study was near to stiffness of dentine or less.

In the current study, the Ni–Cr alloy cast post introduced higher shear stress than glass fiber post. This is due to the contact conditions and difference between the stiffness of the post, which cause amplification of stress magnitudes. These variable stress patterns suggest that increasing the modulus elasticity would increase the interfacial shear stress between the post and cement/dentine interface. The reduction in the interfacial shear stress diminishes the probability of post loosening from dentine as the stress on bonding cement is reduced. Therefore, this study recommended for practitioners utilizing glass fiber post to restore endodontically treated tooth. This is due to that the glass fiber post system can dissipate the interfacial shear stress remarkably from the coronal to the apical regions of the post.

## Conclusions

Glass fiber post reduced the shear stress concentration at post and cement/dentine interfaces compared to Ni–Cr alloy cast post. The reduction in the interfacial shear stress for glass fiber post is advantageous as it likely to be able to resist cyclic loading and reduce the probability of root failure. Therefore, the chosen post with modulus elasticity near to the elastic properties of dentine is preferred.

There was negligible difference in the peak of shear stress when various dental cements were investigated irrespective of the post materials used. Similar trends of shear stress distribution were obtained among tested dental cements.

## Methods

### Solid and FE models preparation

A three-dimensional (3D) model of an adult maxillary central incisor with its surrounding cortical and cancellous bones was developed using a computed tomography (CT) scan. The 3D model was constructed along with their surrounding cortical and cancellous bones using Mimics software (Materialise NV, Belgium) and Hounsfield’s Unit. A periodontal ligament (PDL) was modelled based on the tooth root, with a thickness of 0.25 mm (Madfa et al. 2014), and it was subtracted from the volume of the cortical and cancellous bone. The restorative components of endodontically treated teeth were modelled based on the geometry of the root using a ‘Solid Works’ (Dassault Systèmes, USA). The dimension of each component was based on the data from previous studies (Pegoretti et al. 2002; Asmussen et al. 2005; Boschian Pest et al. 2006; Santos et al. 2009; Abu Kasim et al. 2011; Madfa et al. 2014).

### Finite element analysis

This study used four-node first-order linear tetrahedral solid elements (C3D4). These C3D4s used fine mesh to obtain accurate results because the constant stress on the tetrahedral elements exhibited low convergence. The nodes along the bottom line of the model, referred to as alveolar bone, were fixed in all degrees of freedom (Yang et al. 2001). All components were assumed to be perfectly bonded without any gaps between the components. An oblique load of 100 N, angled at 45°, to simulate the masticatory force was chosen (Madfa et al. 2014). All forces were applied on the aforementioned area as distributed pressure. Any stresses that are likely to be introduced during the endodontic treatment were neglected. The elastic properties of the geometric model parts are shown in Tables 2, 3.

**Table 2:** Mechanical properties of isotropic materials

Material	Elastic modulus (MPa)	Poisson’s ratio
Cortical bone	13,700	0.3
Cancellous bone	1370	0.3
Dentin	18,600	0.32
PDL	0.069	0.45
Porcelain	69,000	0.28
Gutta-percha	140	0.45
Composite resin	12,000	0.33
Ni-Cr alloy	200,000	0.33
Zinc phosphate cement	22,400	0.35
Glass ionomer cement	4000	0.35
Panavia^™^ F	18,600	0.28
Superbond C&B resin cement	1800	0.31

**Table 3:** Mechanical properties of orthotropic materials

Property	Glass fiber post
Ex (MPa)	37,000
Ey (MPa)	9500
Ez (MPa)	9500
Vxy	0.27
Vxz	0.34
Vyz	0.27
Gxy	3100
Gxz	3500
Gyz	3100

Eight 3D finite element models of a maxillary central incisor restored as follows:

1. Glass fiber post cemented with zinc phosphate cement (FP/ZP cement).
2. Glass fiber post cemented with Panavia™ resin cement (FP/Panavia™ cement).
3. Glass fiber post cemented with superbond C&B resin cement (FP/C&B cement).
4. Glass fiber post cemented with glass ionomer cement (FP/GI cement).
5. Ni–Cr alloy cast post cemented with zinc phosphate cement (CP/ZP cement).
6. Ni–Cr alloy cast post cemented with Panavia™ resin cement (CP/Panavia™ cement).
7. Ni–Cr alloy cast post cemented with superbond C&B resin cement (CP/C&B cement).
8. Ni–Cr alloy cast post cemented with glass ionomer cement (CP/GI cement).

The distribution of shear stress was evaluated at the dental post and cement/dentine interfaces using ABAQUS/CAE software, Professional Version. The interfacial shear stress was calculated along the AB path from coronal to apical regions of the dental post and cement/dentine. Interfacial shear stress (τ_*xy*_, τ_*xz*_, and τ_*yz*_) results were virtually mapped onto AB path at the post and cement/dentine interfaces. The computed interfacial stress (τ_*xy*_, τ_*xz*_, and τ_*yz*_) results along the AB path were then analyzed.
